# Positive and negative functions of B lymphocytes in tumors

**DOI:** 10.18632/oncotarget.10094

**Published:** 2016-06-15

**Authors:** Meng Shen, Qian Sun, Jian Wang, Wei Pan, Xiubao Ren

**Affiliations:** ^1^ Department of Immunology, Tianjin Medical University Cancer Institute and Hospital, Tianjin, China; ^2^ Department of Biotherapy, Tianjin Medical University Cancer Institute and Hospital, Tianjin, China; ^3^ National Clinical Research Center of Cancer, Tianjin, China; ^4^ Key Laboratory of Cancer Immunology and Biotherapy, Tianjin, China

**Keywords:** B lymphocytes, regulatory B cells, tumor-infiltrating B cells, tumor, tumor immunotherapy

## Abstract

Accumulating evidence indicated that B lymphocytes exerted complex functions in tumor immunity. On the one hand, B lymphocytes can inhibit tumor development through antibody generation, antigen presentation, tumor tissue interaction, and direct killing. On the other hand, B lymphocytes have tumor-promoting functions. A typical type of B lymphocytes, termed regulatory B cells, is confirmed to attenuate immune response in a tumor environment. In this paper, we summarize the current understanding of B-cell functions in tumor immunology, which may shed light on potential therapeutic strategies against cancer.

## INTRODUCTION

B cells originate from hematopoietic stem cells. After recognizing antigen (Ag), naive B cells enter the primary follicles of lymph nodes or other lymphoid tissues, undergo extensive proliferation, form germinal centers (GCs), and class switch to immunoglobulin (Ig) G, IgA, or IgE. In the GCs, some of the B cells further differentiate into plasma cells (PC), which produce high-affinity antibodies (Abs) [1]. Naive B cells are regulated by numerous signals. Several well-studied signals include B cell receptor (BCR) cross-linking by Ag [2] and stimulation to Toll-like receptor (TLR) expression [3]. Also involved in the process are other novel factors, including PU.1, Spi-B, and Spi-C [4]; ARS-interacting multifunctional protein 1 [5]; B-cell activating factor (BAFF) [6]; and CD137 with its ligands [7].

Numerous studies and clinical trials have described both the positive and negative functions of B lymphocytes in a tumor environment. Given that B cells are considered an integral component of the adaptive immune system, their complicated functions will prominently affect anti-tumor response and be widely used for different clinical applications. This review focuses on the functions of B cells in malignancy and refers to the prospect of targeting B lymphocytes to influence the clinical treatment of tumors in the future.

## POSITIVE EFFECT OF B LYMPHOCYTES ON THE IMMUNE SYSTEM

### Working as Ab generator

After recognizing Ags, some B cells initiate three steps, namely, proliferation, class switch recombination, and PC differentiation. These processes are required for Ab production. At the same time, a large portion of infiltrating B cells are resting, IgM-expressing, or not terminally differentiated into PCs. Abs offer great benefits to anti-tumor immunity when interacting with Ags. For example, the Abs secreted by B cells may bind to tumor Ags and amplify the adaptive immune response in triple-negative breast cancer [8]. Carmi et al. [9] demonstrated that allogeneic IgG combined with dendritic cell (DC) stimuli induces the anti-tumor response of T cells, which implies a new area of B-cell functions as inhibition of tumor development. Both administrations of DCs loaded with allogeneic-IgG-coated tumor cells and intratumoral injection of allogeneic IgG in combination with DC stimuli can result in tumor eradication in several murine cancer models. Similar benefits are also found in lung cancer patients, thus indicating the positive clinical relevance with B-cell combination treatment.

The Ab-based therapy of tumors is commonly applied in patients with hematological malignancies and solid tumors through direct or indirect mechanisms. The therapeutic functions can be emphasized by targeting certain surface Ags of lymphocytes (CD40 [10,11], CD20 [12–14], CD19 [15, 16], CD73 [17, 18]), mediating immune checkpoints (CTLA-4 [19], PD-1 [20, 21]), blocking the bind of specific ligands, perturbing the signaling pathways (EGFR [22, 23], HER2 [24, 25]), and other direct-targeted functions. In combination with conventional cancer therapies, Ab-based therapies that target these factors can stimulate anti-tumor response and improve clinical efficacy. More clinical trials should be further performed to clarify the prognostic and therapeutic values of monoclonal Abs in cancer treatment.

### Working as Ag-presenting cells (APCs)

B cells also can promote anti-tumor immunity through providing Ags to both CD4^+^ and CD8^+^ T cells [26] or through cross-presentation Ags to other APCs [27]. B cells differ from DCs, that is, DCs appear to be vital to initial T-cell priming, whereas B cells may promote T-cell expansion and memory formation [1]. B cells are abundant in circulating blood, and are home to secondary lymphoid organs when administered intravenously [28]. Further studies have indicated that B cells remain more potent than DCs even at a later time when circulating effector T cells decline; hence, B cells are generally superior to DCs when eliciting a recall response [29]. Under a tumor environment, B cells may thus serve as local APCs to sustain the survival and proliferation of tumor-infiltrating T cells.

In high-grade epithelial ovarian cancers (EOCs) patients, CD20^+^ infiltrates are strongly associated with all three T-cell subsets (CD3, CD4, and CD8), as well as T-cell differentiation markers TIA-1, Granzyme B, and FoxP3. Consistent with this, low numbers of CD1a^+^ DCs are also delivered. In this regard, we speculate that CD20^+^ B cells can serve as alternative APCs in a tumor environment [30]. This speculation fits well with the observed co-localization of tumor-infiltrating B cells (TIL-Bs) and CD8^+^ T cells in EOCs, as well as in non-small cell lung carcinoma (NSCLC) [31] and cervical cancer [32]. Within the central nervous system, B cells are also recruited into the brain tumor microenvironment; they then enhance the clonal expansion of tumor-specific T cells and promote T-cell-mediated tumor regression by serving as APCs [33]. Milne et al. discovered that CD20^+^ infiltrates are associated with increased disease-specific survival in EOCs [30]. All these studies have indicated that B cells may act as therapeutic APCs to increase the efficacy of immunotherapeutic strategies for cancer.

### Working as cytokine producer

B cells have recently been found to release interleukin (IL), interferon (IFN), and other cytokines that can stimulate anti-tumor immunity. By producing these cytokines, B cells can interplay with other immunocytes, such as T cells, DCs, macrophages, and natural killer (NK) cells and further influence their functions. The function of B cells as a cytokine producer was first observed in the ROHA-9 cell line, which is an Epstein-Barr virus (EBV)-transformed human B cell line, that could secrete IL-1 and lead to enhanced response of human T cells to concanavalin A [27]. A few studies have been conducted about B cells as cytokine producer in anti-tumor response, but several reports have focused on the interactions between B-cells and other cells by releasing immuno-regulating cytokines, which are speculated to be involved in tumor suppression. For example, the main cytokines produced by naive B cells are the chemokines CCL22 and CCLl7. CCL22, a chemokine expressed mostly by activated B cells and DCs, is referred to induce chemotactic migration of activated T cells by interacting with the specific receptor CCR4 [34]. Other reports also have revealed that B cells with EBV infection can induce the expression of CCL17 and CCL22, and therefore, play vital roles in attracting Th2 cells and regulatory T cells (Tregs). Such B cells also upregulate the expression of CCL3, CCL4, and CCL5, which are all known to attract Th1 and cytotoxic T cells [35]. In response to IL-33, B1b cells can produce significant amounts of macrophage inflammatory protein-1a, granulocyte-macrophage colony-stimulating factor, and vascular endothelial growth factor, thus resulting in the recruitment and growth of monocytes and granulocytes [36]. The above studies have shown that B cells can contribute great benefits to immune response by producing of several cytokines.

### Working as connector with tumor tissues

TIL-Bs represent another research-worthy aspect of the B-cell response to cancer. Several studies on human tumors (e.g., breast, melanoma, and lung cancers) have suggested an improved tumor control in the presence of B cells. Andrea et al. revealed that an increased amount of B cells in cutaneous melanoma can predict long survival and good prognosis [37]. In another work [38], the adoptive transfer of the B cells from the wild-type mice into the B-cell linker protein-deficient mice can attenuate B16F10 melanoma growth. Within the tumor microenvironment, increasing numbers of B and T cells infiltrating into tumors and large percentages of INF-γ- and tumor necrosis factor (TNF) -α-producing tumor-infiltrating T cells in the transferred mice are observed. As for humans, Alexander et al. [39] analyzed the characterization of B cell subsets in colorectal cancer (CRC) patients and found that the B-cell infiltrate of primary CRC is characterized by an accumulation of memory B cells or PCs, which suggests of a specific immune response against the tumor. However, other reports have conversely referred that advanced tumors and metastases are infiltrated by a considerable number of Bregs, which may exert the opposite functions compared with this type of TIL-Bs. As a result, the inner properties and complex roles played by different types of B cells merit further.

In summary, TIL-Bs are prevalently studied in murine and human cancers, and sometimes positively correlate with favorable clinical outcomes. These findings provide a clear rationale for understanding the mechanistic properties of TIL-Bs in anti-tumor response.

### Working as direct killer

The direct cytotoxicity of B cells in killing immuno-inhibitory cells and tumor cells has also been established. Several lines of evidence indicate that some B lymphocytes can express the death-inducing molecule Fas ligand (FasL) and kill cells directly. The expression of FasL by B cells was first reported in 1996 [40]. Upon activation, mouse B cells lead to FasL expression and then kill Fas-expressing cells, such as tumor cells [41]. In fact, TIL-Bs can also directly kill tumor cells through Ab-dependent mechanisms. In colon adenocarcinomas and mammary murine cancer models, the efficacy of anti-DR5 (DR5: a type of tumor-associated death-inducing receptors) therapy is completely abrogated in mice that are deficient of B cells. These data further indicated that Ab-mediated targeting of DR5 can trigger tumor cell apoptosis in established tumors in a B-cell-dependent manner, thereby providing the first direct evidence of the critical functions of B cells in tumor cell apoptosis [42]. As for human beings, B cells induced by IL-21 can secrete granzyme B [43], which has already been proven to up-regulate the direct cytotoxicity against tumor cells in murine models [44]. However, B cells from human and mice may differ in terms of generation and immunological function, and these differences require close attention.

**Figure 1:**
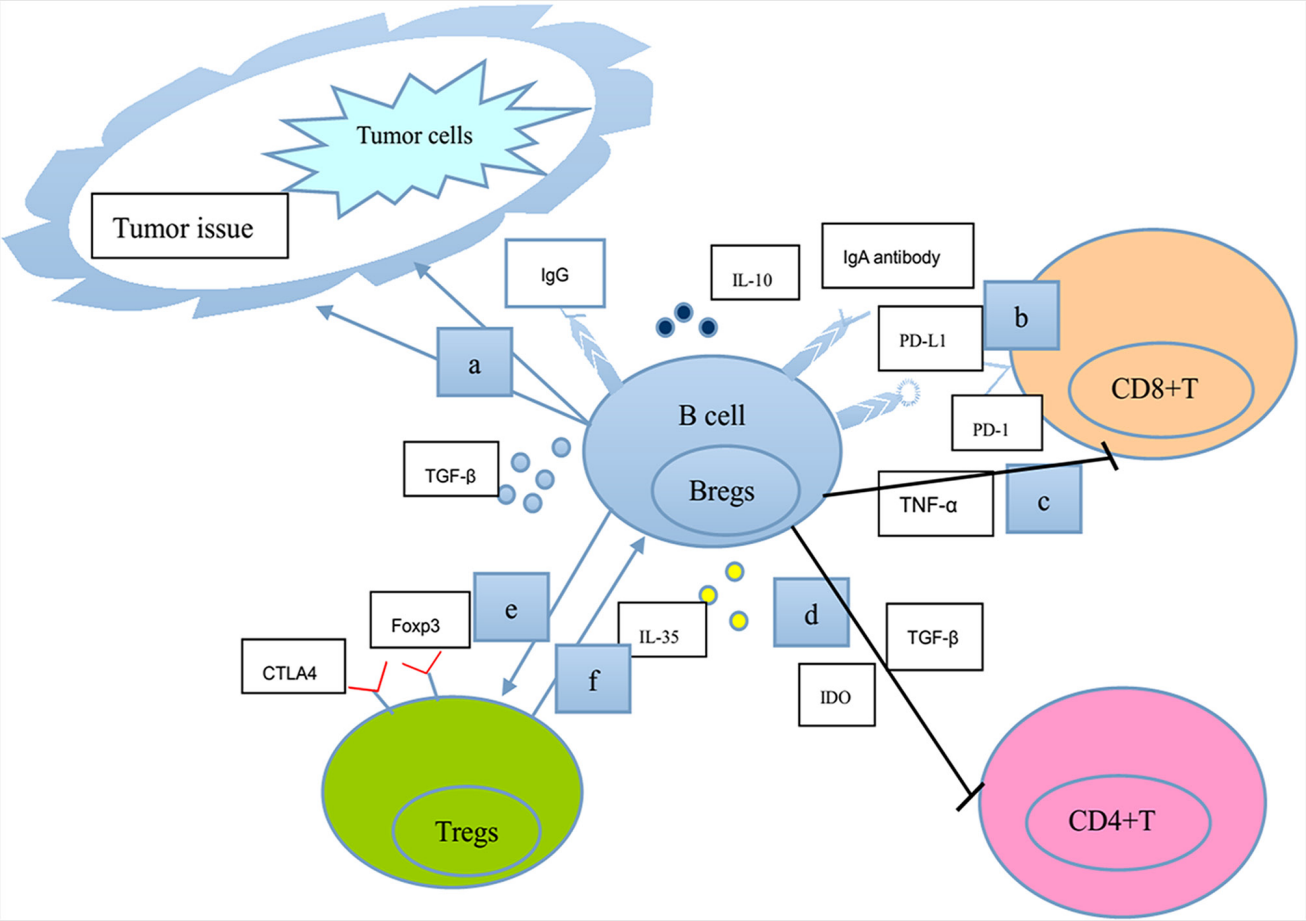
A schematic model shows our current understanding of the negative functions of B lymphocytes in tumor immunity **a.** TGF-β-secreting Bregs affect EMT in tumor tissues directly or in cooperation with Ras or Wnt signaling pathways. **b.** IgA-producing plasmocytes express IL-10, Fas-L, and PD-L1 (the ligand for the T-cell receptor), thus inhibiting T-cell-dependent tumor eradication. **c.** Bregs serve as a significant cellular source of TNF-α to limit immune surveillance by CD8^+^T cells. **d.** Bregs induce the anergy and apoptosis of CD4^+^ T cells through the production of TGF-β and IDO. **e.** Bregs enhance the expression of Foxp3 and CTLA-4 (markers for the suppressive ability of Tregs function) in Tregs through cell-to-cell contact. **f.** CD4^+^CD25^+^Tregs induce the expansion of B10 cells. Consequently, B lymphocytes could perform negative functions in the regulation of many processes associated with tumor immunity.

## NEGATIVE EFFECT OF B LYMPHOCYTES ON THE IMMUNE SYSTEM

In addition to the B-cell functions of inhibiting tumor development, compelling evidence suggests that B cells exert certain functions in suppressing immune response through several ways, with Bregs being the dominant element [45]. Bregs have gained prominence in attenuating immune response in malignant cancers [46, 47], infectious diseases [48], and autoimmune diseases [49]. Moreover, B lymphocytes can work as inhibitory effectors by interacting with tumor tissues and certain types of lymphocytes, such as T cells, APCs, Tregs and myeloid-derived suppressor cells (MDSCs).

### B lymphocytes performing immunosuppressive functions by working as Bregs

Bregs are induced mainly through the BCR pathway, CD40/CD40L, TLR, or BAFF-signaling pathways. In fact, Bregs activation may chiefly involve the TLR instead of the BCR pathway [50]. Other novel molecules are also involved in the process. Recently, Rosser et al. indicated that gut microbiota-driven IL-1β and IL-6 can directly promote the differentiation of Bregs and IL-10 secretion [51]. In addition, a regulatory molecule termed as semaphorin3A [52] induces distinct populations of Bregs and promotes their conversion into “‘i35′-Bregs” both in mice and humans [53]. For transforming growth factor (TGF)-β-secreting Bregs, naive B cells can capture glioma cell-derived placenta growth factor and be differentiated into TGF-β positive Bregs [54]. Esophageal cancer-derived microvesicles exert the same functions [55]. Accumulating evidence shows that activated Bregs can down-regulate the immune response through multiple pathways.

### Bregs suppressing anti-tumor immunity by secreting certain cytokines (IL-10, TGF-β, IL-35)

#### IL-10-secreting Bregs

The concept of a regulatory B cell subset (Bregs) has recently emerged, which involves IL-10 as the main feature for immune suppression. IL-10-secreting B cells (B10 cells) can secrete the inhibitory cytokine IL-10, which possibly has the strongest inhibitory effect on innate immunity. B10 cells can inhibit the differentiation of Th0 cells to Th1 and Th2 cells, as well as suppress the proliferation of T cells [56]. CD19^+^IgM^+^CD27^+^ memory and CD19^+^CD24^hi^CD38^hi^ transitional B-cell subsets can suppress the proliferation of autologous CD4^+^ T cells and IFN-γ production. Both processes rely on IL-10 secretion and cell-to-cell contact [57]. A new wave of researches is beginning to shed light on the functions of Bregs in cancer patients. DiLillo et al. found that chronic lymphocytic leukemia cells resembled Bregs in their phenotype and IL-10 secretion, serving as the first indication that human Bregs may incur malignant expansion [58]. Clinical studies have further demonstrated that the CD19^+^IL-10^+^ Bregs in hepatocellular carcinoma (HCC) patients are significantly lower than those in healthy donors and patients with chronic hepatitis B infection before surgery, but they dramatically increase and remain high after surgery (about seven days after surgery, *p* < 0.05) [59]. In NSCLC patients [60], the frequency and absolute number of B10 cells are significantly elevated and further associated with the clinical stage. The number of B10 cells of stage IV NSCLC patients is significantly elevated compared with that of healthy donors, and stage II and stage III patients. Increased B-cell subset may lead to poor clinical prognosis in NSCLC. Similar results are also found in ovarian cancer [61]. The population of B10 cells is preferentially enriched in ascites, and their frequency is positively correlated with ovarian cancer severity. Stage III ovarian cancer patients have higher frequencies of IL-10^+^ B cells than stage II patients, both in the peripheral blood and ascites. Thus, Bregs contribute to the impaired anti-tumor immunity in ovarian cancer patients. In tongue squamous cell carcinoma, the increased frequency of Bregs in tumor microenvironment is shown to be related to Tregs and similarly predicts worse survival [62]. These reports have demonstrated an additional regulatory mechanism in the tumor microenvironment, which utilizes IL-10^+^ B cells.

**Figure 2:**
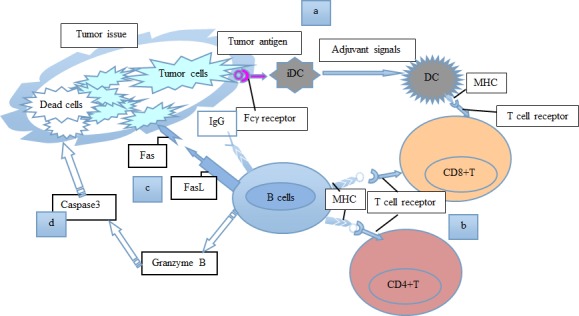
A schematic model shows our current understanding of the positive roles of B lymphocytes in tumor immunity **a.** Allogeneic B cells secrete IgG antibodies to recognize surface molecules on tumor cells, activate DCs, and induce the cell-killing activity of CD8^+^ T cells. **b.** B cells function as APCs for CD4^+^ and CD8^+^ T cells. **c.** B cells could express the death-inducing molecule FasL, and kill tumor cells through Fas-FasL connections. **d.** B cells could secrete granzyme B, to cause caspase3 activation and tumor cell apoptosis. Consequently, B lymphocytes perform positive functions in the regulation of many processes associated with tumor immunity.

### IL-35-secreting Bregs

Bregs are regarded as a vital source of IL-35. As the newest IL-12 family member, IL-35 can suppress T-cell proliferation and function *in vitro* and *in vivo*. A deficient IL-35 production resulted in the high activation of macrophages and inflammatory T cells [63]. Some connections may exist between IL-35- and IL-10-secreting B cells. Some reports have found that IL-35 can induce Bregs and promote their conversion to the subsets that produce IL-35 and IL-10 [53, 64]. These findings may emphasize the possible connections between the two types of Bregs, which needs to be further considered in tumor progression.

A few studies have focused on the immunosuppressive function of IL-35-secreting Bregs in tumor promotion. Zhang et al. found that IL-35 is an independent prognostic factor and a therapeutic target for nasopharyngeal carcinoma [65]. The expressions of two IL-35 subunits are significantly associated with the advancement of tumor stage and indicated unfavorable prognosis (*p* < 0.05). The over-expression of IL-35 is also correlated with the genesis of gastric cancer through promoting the growth and apoptosis of cancer cells [66]. During the development of pancreatic neoplasia [67], the pro-tumorigenic effect of B cells is found to be mediated by IL-35 expression through a mechanism involving IL-35-mediated stimulation of tumor cell proliferation. In B-cell-deficient mice, the neoplasms growth harboring oncogenic Kras is significantly compromised, and the deficiency can be rescued by the reconstitution of a CD1d^hi^CD5^+^ B-cell subset which can produce IL-35. These results point to the close connections between IL-35-secreting Bregs and tumor cells, and identify a rationale for exploring B-cell-based approaches for treating malignancies.

### TGF-β-secreting Bregs

In addition to IL-10- and IL-35-secreting Bregs, TGF-β-secreting Bregs have attracted significant attention. For example, glioma-derived ADAM10 can induce TGF-β expression in the B cells, and convert naive B cells to Bregs. These B cells are demonstrated to suppress the proliferation of CD8^+^ T cell and induce Tregs. [68]. By secreting TGF-β, Bregs can promote the accumulation of the mesenchymal marker vimentin in the process of epithelial-mesenchymal transition (EMT) in tumor tissues [69]. A study has found that TGF-β, in cooperation with Ras signals, can induce EMT during the progression of epithelial tumors [70]. TGF-β also can work together with Wnt-signaling pathways in CRC through FOXQ1 mediation [71]. All these facts indicate the potential immunosuppressive function of TGF-β-secreting Bregs.

### Bregs suppressing anti-tumor immunity by affecting other immunocytes

#### By affecting the function of T cells

An experimental application infers that co-culturing Bregs with autologous stimulated CD4^+^ T cells can result in significantly reduced proliferative capacity of the latter cells [72]. A study also has shown that Bregs could induce the anergy and apoptosis of CD4^+^ T cells through producing TGF-β and indoleamine 2, 3-dioxygenase [73]. In 7, 12-dimethylbenz [α]anthracene/terephthalic acid-induced squamous carcinogenesis mice models, Bregs are a significant cellular source of TNF-α and act as important effector cells for TNF-α-mediated promotion of cancer development. Bregs can limit immune surveillance by CD8^+^ T cells [74]. As a result, Bregs may inhibit T cell proliferation through cell-to-cell contact, thereby leading to anergy or apoptosis [75]. Moreover, B10 cells from the ascites of ovarian tumor [61] can suppress the IFN-γ production of CD8^+^ T cells. When co-cultured CD8^+^ T cells with autologous blood B cells or ascitic B cells, the co-cultured group demonstrates significantly decreased IFN-γ production. The suppression was found in part mediated by IL-10. All these findings show that Bregs can impair anti-tumor response through affecting the functions of T cells.

### By affecting the function of other immunocytes beyond T cells

In addition to their immunosuppressive function in T cells, Bregs employ other mechanisms in targeting other types of immunocytes such as Tregs, MDSCs, DCs, macrophages and monocytes [76, 77, 78].

#### Tregs

As reported, Bregs facilitate the earlier stage of the recruitment of Tregs in autoimmune disorders [50]. In one study, thymic CD19^+^CD5^+^CD1d^hi^IL-10^+^ Bregs perform critical functions in the maintenance of immune homeostasis. When co-cultured with CD4^+^ T cells, this population of B cells supported the maintenance of CD4^+^ Foxp3^+^ Tregs *in vitro*. The transfer of these B cells into CD19^−/−^ mice results in significantly up-regulated numbers of CD4^+^Foxp3^+^ Tregs in the thymus, spleen, and lymph nodes [79]. As for tumor environment, several studies have focused on the interplay between Bregs and Tregs. Recently, cytological experiments [62] have indicated that induced by tongue squamous cell carcinoma cells, B cells can convert CD4^+^CD25^−^ T cells into Tregs. In the ascites of ovarian cancer patients, the frequencies of IL-10^+^ B cells are also positively correlated with the frequencies of CD4^+^Foxp3^+^ Tregs [61]. In another study, when targeting Bregs by Lipoxin A4, the number of Tregs decreases in the draining lymph nodes and tumor tissues [80], thereby emphasizing the important effect of Bregs on inducing the frequency and distribution of Tregs. Kessel et al. also demonstrated that human Bregs can enhance the expression of Foxp3 and CTLA-4 (markers for the suppressive capability of the function of Tregs) in Tregs through cell-to-cell contact [72]. Currently, co-culturing stimulated Bregs with Tregs may result in a significant increase in Foxp3 levels in comparison with Tregs cultured alone (5.93 ± 0.18 *vs.* 4.38 ± 0.11, *p* < 0.05). No difference is observed when co-cultured with non-Breg cells (4.22 ± 0.28 *vs.* 4.44 ± 0.23, *p* > 0.05) [52]. Conversely, CD4^+^CD25^+^ Tregs can also induce the expansion of B10 cells [81], thus facilitating a vital interaction between the two cells. A unique Bregs subset called tumor-evoked Bregs (tBregs) is discovered in breast cancer. These cells can protect the metastasizing cancer cells from immune effector cells by inducing immune suppression, which is mediated by Tregs [82]. In addition, clinical studies have further revealed that both circulating Tregs and Bregs increase after HCC surgery [59]. Therefore, a comprehensive adjuvant immunotherapy targeting on depleting Tregs and Bregs may be beneficial for the improved prognosis of post-surgery HCC patients. This prospect highlights the functions of Bregs in the tumor promotion and metastasis.

### MDSCs

MDSCs are a heterogeneous population of immature myeloid cells reported to promote immunosuppressive response and facilitate tumor metastasis and invasion. Prior study [60] has shown that the frequency of IL-10 producing B cells was significantly positively correlated with the frequency of CD14^+^HLA^−^DR^low/−^ MDSCs (*r* = 0.3948, *p* < 0.05) during the progression of NSCLC. In addition, the immunosuppressive and prometastatic functions of MDSCs partly rely on the education from tBregs [78], partly through TgfbR1/TgfbR2 signaling. The tBregs deficiency in MDSCs is sufficient to disable their suppressive function and to block metastasis. Herein, the potential connections between Bregs and MDSCs can provide clinical benefits in anti-tumor response.

### Bregs suppressing anti-tumor immunity by directly interacting with malignant cells

Bregs have recently been found to promote the growth and invasiveness of HCC by directly interacting with liver cancer cells through the CD40/CD154 signaling pathway [47], thus providing a novel method for suppressing the anti-tumor process. Zhou et al. showed that the increasing frequencies of Bregs seem to be modulated directly by tumor cells in patients with lung cancer and thus, indicate the direct interaction between Bregs and malignant cells [46].

As a result, activated Bregs can directly or indirectly target immunocytes and tumor cells to inhibit immune responses. In this case, the down-regulation of Bregs may become a therapeutic method for treating tumors.

### B lymphocytes performing immunosuppressive functions by interacting with T cells

Zhang et al. found that the absence of B cells leads to reduced tumor growth, which is accompanied by increased infiltration of T and NK cells [83]. The observation describes that B cells may attenuate anti-tumor cytotoxic T cells responses and potentiate the local expansion of CD4^+^FoxP3^+^ Tregs in murine tumor environment. They also highlighted that B cells can inhibit T-cell anti-tumor response through Ag non-specific mechanisms. In another report utilizing murine EMT-6 mammary tumor models [84], wide-type mice demonstrates extensive B cell infiltration into the tumor sites and reduced infiltration of CD8^+^ T cells and CD49^+^ NK cells relative to B-cell-deficient mice. Co-culturing of EMT-6 tumor cells with naive B cells *ex vivo* generates similar immunosuppressive phenotypes with TIL-B cells, thus resulting in profound inhibition of T-cell anti-tumor reactions. In a study using three mouse prostate cancer models, IgA-producing plasmocytes express programmed death ligand 1 (PD-L1), IL-10, and Fas-L, and thus inhibited T-cell-dependent tumor eradication [85]. Another study similarly indicated that IgA^+^ plasmocytes may induce CD8^+^ T-cell exhaustion [86]. In return, the depletion of B cells may result in positive effects on promoting some types of T cells. In a mouse model of cervical cancer [87], B cells are accumulated in the draining lymph nodes, but they do not exhibit a classical Bregs phetotype or secrete IL-10 *in vitro*. These B cells express markers, such as PD-L1 and CD39, which may play a regulatory role by directly inhibiting the T-cell response. Similarly, the tumor growth is indeed significantly impaired in the B-cell-deficient mice. This study also demonstrates that B cells can participate in establishing an immunosuppressive tumor environment by impairing T cell-dependent immunity in a direct way. Affara et al. found that the depletion of B cells reprograms macrophages to recruit CD8^+^ T cells in squamous carcinomas (SCCs). B-cell-deficient mice present reduced ability to support SCC growth and improved chemo-responsiveness. The responsiveness is further analyzed to be dependent on altered chemokine expression by macrophages that foster tumor infiltration of activated CD8^+^ T cells. This finding reveals that B cells may deregulate T-cell infiltration in an indirect way [88]. Another study [89] has shown that B-cell depletion can enhance the establishment of CD49b^+^ T-bet^+^ resting memory T helper cells in the bone marrow (BM) and that B-cell transplantation conversely suppresses it. By using B-cell-depleted or B-cell-deficient mice, they suggested that B cells are a negative regulator to generate CD4^+^ T cell memory in the BM and contribute to a quantitative balance of the commitment to effector T helper cells and resting memory CD4^+^ T cell lineage.

### B lymphocytes performing immunosuppressive functions by correlating with tumor tissues

Several preliminary and clinical experiments have revealed that many tumors are heavily infiltrated with B cells, including breast cancer [90], prostate cancer [91], and CRC [39]. In contrast to the anti-tumor functions of TIL-B cells mentioned above, the immunosuppressive properties of TIL-B cells have been commonly studied. CD19^+^B cells isolated from tumors present a considerably greater suppressive effect on CD4^+^T-cell proliferation in comparison with the cells isolated from the spleen of tumor-bearing or non-tumor-bearing animal models [83]. Result confirms that B cells can suppress immune response in connection with tumors. By comparing TIL-B cells with splenic B cells, Zhang et al. [84] found that TIL-B cells acquire increased expression of immunosuppressive ligands, such as LAP/TGF-β1, CD80, CD86 and PD-L1, therefore leading to enhanced inhibitory activity against CD4^+^CD25^−^ T, CD8^+^ T and NK cells. Another study on murine melanoma has indicated that under determined conditions, B-1 cells can come into contact with tumor cells and acquire increased metastatic behavior. This phenomenon is associated with the activation of the extracellular signal-regulated kinase pathway in melanoma cells [92]. B-cell lineage in tumor issues and tumor-draining lymph nodes are clonally and functionally related to each other. The physiological relationship between the two sources of B lymphocytes may be relative to tumor-specific immune responses in breast cancer patients [90]. B cells and downstream myeloid-based pathways regulate represented tractable targets for combinatorial therapy in SCC; hence, B-cell depletion may be relatively straightforward for achieving clinical benefits [88]. Moreover, B cells may be positively correlated with the poor prognosis of patients harboring metastatic carcinomas. For example, when recruited by chemokine CXCL13, B cells can produce lymphotoxin to promote the progression of castration-resistant prostate cancer [93], mainly through activating of the IKKa-BMI1 pathway in cancerous prostate stem cells [94]. This finding further indicates that B cells may be a potent biomarker in speculating the prognosis of cancer patients. B cells are also known to induce decreased response to chemotherapy. Recently, Shalapour et al. discovered that B cells can down-regulate the response to low-dose oxaliplatin (an immunogenic chemotherapeutic agent) in mouse prostate cancer models; specifically, B cells can induce immunogenic cell apoptosis to promote tumor-directed cytotoxic lymphocyte activation. Such immunosuppressive B cells are plasmocytes that express IgA, IL-10, and PD-L1, depending mostly on TGF-β receptor signaling [85]. Similarly, B-cell depletion by administrating of anti-CD20 monoclonal Abs can significantly improve the response of patients to chemotherapy [88]. Therefore, we suggest that the elimination or inhibition of TIL-Bs may be crucial to the successful immunotherapy of tumors.

### B lymphocytes performing immunosuppressive functions by secreting tumor-reactive Abs

The effect of naturally arising tumor-reactive Abs on tumor progression has long been controversial. Carmi et al. emphasized the role of such Abs depending on the environmental context and involved cell types [9]. Under certain circumstance, these Abs may promote tumor progression. For example, Andreu et al. described that B cells can secret auto-Abs to promote tumor development through interacting with activating Fcγ receptors on resident and recruited myeloid cells [95]. B cells also perform crucial functions in inducing the epithelial cancer development possibly by initiating Ig deposition into neoplastic tissue paralleling chronic inflammation and pre-malignant progression [96]. Obviously, the immunosuppressive function of B cells merits research attention.

Cancer cell-derived IgG (cancer-IgG) is also involved in the pathogenesis and progression of many cancers. Recently, Liu et al. utilized an RP215 monoclonal Ab to determine cancer-IgG expression in 140 lung adenocarcinoma patients. RP215-positive cells display greater migration and invasion capabilities than RP215-negative cells. A high RP215 immunostaining score is associated significantly with poor prognosis [97]. This report emphasizes the positive connections between cancer-related Ig and the poor prognosis of cancer patients. As described above, a certain type of plasmocytes that expresses IgA can perform immunosuppressive roles in aggressive prostate cancer. These B cells can inhibit oxaliplatin-induced tumor regression. Hence, we can infer that the elimination or inhibition of tumor-infiltrating IgA plasmocytes may be the key to successful immunotherapy, in combination with immunogenic chemotherapeutics, such as oxaliplatin [85].

## PERSPECTIVE AND THERAPEUTIC OPTIONS

Current evidence suggests that B-cell repertoire may perform potent roles in promoting and inhibiting tumor development, which underscores the potential significance of B-cell as a therapeutic target. Recent clinical trials that have targeted B cells, typical Abs or associated cytokines have also lent further support to the idea of B cells serving as crucial regulators of anti-tumor activities. However, the functions of B cells in tumor immunity, and the effect of B-cell malfunctions on oncogenesis and tumor progression should be elucidated thoroughly. An improved understanding of these normal and pathogenic mechanisms will aid in developing of novel and efficient approaches to tumor therapy.

## References

[1] Nelson BH (2010). CD20+ B cells: the other tumor-infiltrating lymphocytes. Journal of immunology.

[2] Xu L, Li G, Wang J, Fan Y, Wan Z, Zhang S, Shaheen S, Li J, Wang L, Yue C, Zhao Y, Wang F, Brzostowski J (2014). Through an ITIM-independent mechanism the FcgammaRIIB blocks B cell activation by disrupting the colocalized microclustering of the B cell receptor and CD19. Journal of immunology.

[3] Ruprecht CR, Lanzavecchia A (2006). Toll-like receptor stimulation as a third signal required for activation of human naive B cells. European journal of immunology.

[4] DeKoter RP, Geadah M, Khoosal S, Xu LS, Thillainadesan G, Torchia J, Chin SS, Garrett-Sinha LA (2010). Regulation of follicular B cell differentiation by the related E26 transformation-specific transcription factors PU 1 Spi-B and Spi-C. Journal of immunology.

[5] Kim MS, Kim TS (2015). Aminoacyl tRNA Synthetase-Interacting Multifunctional Protein 1 acts as a novel B cell-activating factor in vitro and in vivo. Journal of immunology.

[6] Mackay F, Schneider P (2009). Cracking the BAFF code. Nature reviews immunology.

[7] Kienzle G, von Kempis J (2000). CD137 (ILA/4-1BB) expressed by primary human monocytes induces monocyte activation and apoptosis of B lymphocytes. International immunology.

[8] Disis ML, Stanton SE (2015). Triple-negative breast cancer: immune modulation as the new treatment paradigm. American Society of Clinical Oncology educational book / ASCO American Society of Clinical Oncology Meeting.

[9] Carmi Y, Spitzer MH, Linde IL, Burt BM, Prestwood TR, Perlman N, Davidson MG, Kenkel JA, Segal E, Pusapati GV, Bhattacharya N, Engleman EG (2015). Allogeneic IgG combined with dendritic cell stimuli induce antitumour T-cell immunity. Nature.

[10] Hassan SB, Sorensen JF, Olsen BN, Pedersen AE (2014). Anti-CD40-mediated cancer immunotherapy: an update of recent and ongoing clinical trials. Immunopharmacology and immunotoxicology.

[11] Jackaman C, Cornwall S, Graham PT, Nelson DJ (2011). CD40-activated B cells contribute to mesothelioma tumor regression. Immunology and cell biology.

[12] Chen Y, Chen L, Lu Q, Meng Y, Wang C, Wang L, Wang H, Yu X, Zhang Y, Zhao L, Li B, Guo Y (2015). Optimization of anti-CD20 humanized antibody hu8E4 by site-directed mutation based on epitope analysis. Biochemical and biophysical research communications.

[13] Ardeshna KM, Qian W, Smith P, Braganca N, Lowry L, Patrick P, Warden J, Stevens L, Pocock CF, Miall F, Cunningham D, Davies J, Jack A (2014). Rituximab versus a watch-and-wait approach in patients with advanced-stage asymptomatic non-bulky follicular lymphoma: an open-label randomised phase 3 trial. The Lancet oncology.

[14] Shuang YR, Ye DQ, Chen JX, Wu YH, Huang H, Fan GH (2008). Observation of the curative effect of Mabthera in combination with the CHOP regimen in treating invasive B-cell lymphoma: A report of 45 cases. Chinese journal of clinical oncology.

[15] von Muenchow L, Engdahl C, Karjalainen K, Rolink AG (2014). The selection of mature B cells is critically dependent on the expression level of the co-receptor CD19. Immunology letters.

[16] Zugmaier G, Klinger M, Schmidt M, Subklewe M (2015). Clinical overview of anti-CD19 BiTE((R)) and ex vivo data from anti-CD33 BiTE((R)) as examples for retargeting T cells in hematologic malignancies. Molecular immunology.

[17] Gao ZW, Dong K, Zhang HZ (2014). The roles of CD73 in cancer. BioMed research international.

[18] Forte G, Sorrentino R, Montinaro A, Luciano A, Adcock IM, Maiolino P, Arra C, Cicala C, Pinto A, Morello S (2012). Inhibition of CD73 improves B cell-mediated anti-tumor immunity in a mouse model of melanoma. Journal of immunology.

[19] Villaruz LC, Kalyan A, Zarour H, Socinski MA (2014). Immunotherapy in lung cancer. Translational lung cancer research.

[20] Santarpia M, Karachaliou N (2015). Tumor immune microenvironment characterization and response to anti-PD-1 therapy. Cancer biology & medicine.

[21] Teixido C, Karachaliou N, Gonzalez-Cao M, Morales-Espinosa D, Rosell R (2015). Erratum to Assays for predicting and monitoring responses to lung cancer immunotherapy. Cancer biology & medicine.

[22] Hong L, Han Y, Brain L (2014). The role of epidermal growth factor receptor in prognosis and treatment of gastric cancer. Expert review of gastroenterology & hepatology.

[23] Seshacharyulu P, Ponnusamy MP, Haridas D, Jain M, Ganti AK, Batra SK (2012). Targeting the EGFR signaling pathway in cancer therapy. Expert opinion on therapeutic targets.

[24] Lemos LG, Victorino VJ, Herrera AC, Aranome AM, Cecchini AL, Simao AN, Panis C, Cecchini R (2015). Trastuzumab-based chemotherapy modulates systemic redox homeostasis in women with HER2-positive breast cancer. International immunopharmacology.

[25] Schrama D, Reisfeld RA, Becker JC (2006). Antibody targeted drugs as cancer therapeutics. Nature reviews drug discovery.

[26] Parker Harp CR, Archambault AS, Sim J, Ferris ST, Mikesell RJ, Koni PA, Shimoda M, Linington C, Russell JH, Wu GF (2015). B cell antigen presentation is sufficient to drive neuroinflammation in an animal model of multiple sclerosis. Journal of immunology.

[27] Crawford A, Macleod M, Schumacher T, Corlett L, Gray D (2006). Primary T cell expansion and differentiation in vivo requires antigen presentation by B cells. Journal of immunology.

[28] Szeto GL, Van Egeren D, Worku H, Sharei A, Alejandro B, Park C, Frew K, Brefo M, Mao S, Heimann M, Langer R, Jensen K, Irvine DJ (2015). Microfluidic squeezing for intracellular antigen loading in polyclonal B-cells as cellular vaccines. Scientific reports.

[29] Zhang L, Bridle BW, Chen L, Pol J, Spaner D, Boudreau JE, Rosen A, Bassett JD, Lichty BD, Bramson JL, Wan Y (2013). Delivery of viral-vectored vaccines by B cells represents a novel strategy to accelerate CD8(+) T-cell recall responses. Blood.

[30] Milne K, Kobel M, Kalloger SE, Barnes RO, Gao D, Gilks CB, Watson PH, Nelson BH (2009). Systematic analysis of immune infiltrates in high-grade serous ovarian cancer reveals CD20 FoxP3 and TIA-1 as positive prognostic factors. PloS one.

[31] Al-Shibli KI, Donnem T, Al-Saad S, Persson M, Bremnes RM, Busund LT (2008). Prognostic effect of epithelial and stromal lymphocyte infiltration in non-small cell lung cancer. Clinical cancer research.

[32] Nedergaard BS, Ladekarl M, Nyengaard JR, Nielsen K (2008). A comparative study of the cellular immune response in patients with stage IB cervical squamous cell carcinoma. Low numbers of several immune cell subtypes are strongly associated with relapse of disease within 5 years. Gynecologic oncology.

[33] Candolfi M, Curtin JF, Yagiz K, Assi H, Wibowo MK, Alzadeh GE, Foulad D, Muhammad AK, Salehi S, Keech N, Puntel M, Liu C, Sanderson NR (2011). B cells are critical to T-cell-mediated antitumor immunity induced by a combined immune-stimulatory/conditionally cytotoxic therapy for glioblastoma. Neoplasia.

[34] Ghadially H, Ross XL, Kerst C, Dong J, Reske-Kunz AB, Ross R (2005). Differential regulation of CCL22 gene expression in murine dendritic cells and B cells. Journal of immunology.

[35] Yoshie O, Imai T, Nomiyama H (2001). Chemokines in immunity. Advances in immunology.

[36] Ahmed A, Koma MK (2015). Interleukin-33 triggers B1 cell expansion and its release of monocyte/macrophage chemoattractants and growth factors. Scandinavian journal of immunology.

[37] Ladanyi A (2015). Prognostic and predictive significance of immune cells infiltrating cutaneous melanoma. Pigment cell & melanoma research.

[38] Kobayashi T, Hamaguchi Y, Hasegawa M, Fujimoto M, Takehara K, Matsushita T (2014). B cells promote tumor immunity against B16F10 melanoma. The American journal of pathology.

[39] Shimabukuro-Vornhagen A, Schlosser HA, Gryschok L, Malcher J, Wennhold K, Garcia-Marquez M, Herbold T, Neuhaus LS, Becker HJ, Fiedler A, Scherwitz P, Koslowsky T, Hake R (2014). Characterization of tumor-associated B-cell subsets in patients with colorectal cancer. Oncotarget.

[40] Hahne M, Renno T, Schroeter M, Irmler M, French L, Bornard T, MacDonald HR, Tschopp J (1996). Activated B cells express functional Fas ligand. European journal of immunology.

[41] Li Y, Xu DF, Jiang D, Zhao J, Ge JF, Zheng SY (2015). Significance of Fas and FasL protein expression in cardiac carcinoma and local lymph node tissues. International journal of clinical and experimental pathology.

[42] Haynes NM, Hawkins ED, Li M, McLaughlin NM, Hammerling GJ, Schwendener R, Winoto A, Wensky A, Yagita H, Takeda K, Kershaw MH, Darcy PK, Smyth MJ (2010). CD11c+ dendritic cells and B cells contribute to the tumoricidal activity of anti-DR5 antibody therapy in established tumors. Journal of immunology.

[43] Hagn M, Schwesinger E, Ebel V, Sontheimer K, Maier J, Beyer T, Syrovets T, Laumonnier Y, Fabricius D, Simmet T, Jahrsdorfer B (2009). Human B cells secrete granzyme B when recognizing viral antigens in the context of the acute phase cytokine IL-21. Journal of immunology.

[44] Catalan E, Jaime-Sanchez P, Aguilo N, Simon MM, Froelich CJ, Pardo J (2015). Mouse cytotoxic T cell-derived granzyme B activates the mitochondrial cell death pathway in a Bim-dependent fashion. The Journal of biological chemistry.

[45] Horikawa M, Minard-Colin V, Matsushita T, Tedder TF (2011). Regulatory B cell production of IL-10 inhibits lymphoma depletion during CD20 immunotherapy in mice. The Journal of clinical investigation.

[46] Zhou J, Min Z, Zhang D, Wang W, Marincola F, Wang X (2014). Enhanced frequency and potential mechanism of B regulatory cells in patients with lung cancer. Journal of translational medicine.

[47] Shao Y, Lo CM, Ling CC, Liu XB, Ng KT, Chu AC, Ma YY, Li CX, Fan ST, Man K (2014). Regulatory B cells accelerate hepatocellular carcinoma progression via CD40/CD154 signaling pathway. Cancer letters.

[48] Kaltenmeier C, Gawanbacht A, Beyer T, Lindner S, Trzaska T, van der Merwe JA, Harter G, Gruner B, Fabricius D, Lotfi R, Schwarz K, Schutz C, Honig M (2015). CD4+ T cell-derived IL-21 and deprivation of CD40 signaling favor the in vivo development of granzyme B-expressing regulatory B cells in HIV patients. Journal of immunology.

[49] Lin W, Jin L, Chen H, Wu Q, Fei Y, Zheng W, Wang Q, Li P, Li Y, Zhang W, Zhao Y, Zeng X, Zhang F (2014). B cell subsets and dysfunction of regulatory B cells in IgG4-related diseases and primary Sjogren's syndrome: the similarities and differences. Arthritis research & therapy.

[50] Berthelot JM, Jamin C, Amrouche K, Le Goff B, Maugars Y, Youinou P (2013). Regulatory B cells play a key role in immune system balance. Joint bone spine: revue du rhumatisme.

[51] Rosser EC, Oleinika K, Tonon S, Doyle R, Bosma A, Carter NA, Harris KA, Jones SA, Klein N, Mauri C (2014). Regulatory B cells are induced by gut microbiota-driven interleukin-1beta and interleukin-6 production. Nature medicine.

[52] Vadasz Z, Haj T, Kessel A, Toubi E (2013). B-regulatory cells in autoimmunity and immune mediated inflammation. FEBS letters.

[53] Wang RX, Yu CR, Dambuza IM, Mahdi RM, Dolinska MB, Sergeev YV, Wingfield PT, Kim SH, Egwuagu CE (2014). Interleukin-35 induces regulatory B cells that suppress autoimmune disease. Nature medicine.

[54] Han S, Feng S, Ren M, Ma E, Wang X, Xu L, Xu M (2014). Glioma cell-derived placental growth factor induces regulatory B cells. The international journal of biochemistry & cell biology.

[55] Li Y, An J, Huang S, He J, Zhang J (2015). Esophageal cancer-derived microvesicles induce regulatory B cells. Cell biochemistry and function.

[56] Tedder TF (2015). B10 cells: a functionally defined regulatory B cell subset. Journal of immunology.

[57] Khoder A, Sarvaria A, Alsuliman A, Chew C, Sekine T, Cooper N, Mielke S, de Lavallade H, Muftuoglu M, Fernandez Curbelo I, Liu E, Muraro PA, Alousi A (2014). Regulatory B cells are enriched within the IgM memory and transitional subsets in healthy donors but are deficient in chronic GVHD. Blood.

[58] DiLillo DJ, Weinberg JB, Yoshizaki A, Horikawa M, Bryant JM, Iwata Y, Matsushita T, Matta KM, Chen Y, Venturi GM, Russo G, Gockerman JP, Moore JO (2013). Chronic lymphocytic leukemia and regulatory B cells share IL-10 competence and immunosuppressive function. Leukemia.

[59] Chen T, Song D, Min Z, Wang X, Gu Y, Wei B, Yao J, Chen K, Jiang Z, Xie H, Zhou L, Zheng S (2012). Perioperative dynamic alterations in peripheral regulatory T and B cells in patients with hepatocellular carcinoma. Journal of translational medicine.

[60] Liu J, Wang H, Yu Q, Zheng S, Jiang Y, Liu Y, Yuan G, Qiu L (2016). Aberrant frequency of IL-10-producing B cells and its association with Treg and MDSC cells in non small cell lung carcinoma patients. Human immunology.

[61] Wei X, Jin Y, Tian Y, Zhang H, Wu J, Lu W, Lu X (2016). Regulatory B cells contribute to the impaired antitumor immunity in ovarian cancer patients. Tumour biology.

[62] Zhou X, Su YX, Lao XM, Liang YJ, Liao GQ (2016). CD19(+)IL-10(+) regulatory B cells affect survival of tongue squamous cell carcinoma patients and induce resting CD4(+) T cells to CD4(+)Foxp3(+) regulatory T cells. Oral oncology.

[63] Shen P, Roch T, Lampropoulou V, O'Connor RA, Stervbo U, Hilgenberg E, Ries S, Dang VD, Jaimes Y, Daridon C, Li R, Jouneau L, Boudinot P (2014). IL-35-producing B cells are critical regulators of immunity during autoimmune and infectious diseases. Nature.

[64] Tedder TF, Leonard WJ (2014). Autoimmunity: regulatory B cells--IL-35 and IL-21 regulate the regulators. Nature reviews rheumatology.

[65] Zhang Y, Sun H, Wu H, Tan Q, Xiang K (2015). Interleukin 35 is an independent prognostic factor and a therapeutic target for nasopharyngeal carcinoma. Contemporary oncology.

[66] Fan YG, Zhai JM, Wang W, Feng B, Yao GL, An YH, Zeng C (2015). IL-35 over-expression is associated with genesis of gastric cancer. Asian Pacific journal of cancer prevention.

[67] Pylayeva-Gupta Y, Das S, Handler JS, Hajdu CH, Coffre M, Koralov SB, Bar-Sagi D (2016). IL35-producing B cells promote the development of pancreatic neoplasia. Cancer discovery.

[68] Ye ZP, He HY, Wang H, Li WS, Luo L, Huang ZC, Guo Y (2014). Glioma-derived ADAM10 induces regulatory B cells to suppress CD8+ T cells. PloS one.

[69] Jensen-Jarolim E, Fazekas J, Singer J, Hofstetter G, Oida K, Matsuda H, Tanaka A (2015). Crosstalk of carcinoembryonic antigen and transforming growth factor-beta via their receptors: comparing human and canine cancer. Cancer immunology immunotherapy.

[70] Saitoh M, Endo K, Furuya S, Minami M, Fukasawa A, Imamura T, Miyazawa K (2016). STAT3 integrates cooperative Ras and TGF-beta signals that induce Snail expression. Oncogene.

[71] Peng X, Luo Z, Kang Q, Deng D, Wang Q, Peng H, Wang S, Wei Z (2015). FOXQ1 mediates the crosstalk between TGF-beta and Wnt signaling pathways in the progression of colorectal cancer. Cancer biology & therapy.

[72] Kessel A, Haj T, Peri R, Snir A, Melamed D, Sabo E, Toubi E (2012). Human CD19(+)CD25(high) B regulatory cells suppress proliferation of CD4(+) T cells and enhance Foxp3 and CTLA-4 expression in T-regulatory cells. Autoimmunity reviews.

[73] Nouel A, Pochard P, Simon Q, Segalen I, Le Meur Y, Pers JO, Hillion S (2015). B-Cells induce regulatory T cells through TGF-beta/IDO production in a CTLA-4 dependent manner. Journal of autoimmunity.

[74] Schioppa T, Moore R, Thompson RG, Rosser EC, Kulbe H, Nedospasov S, Mauri C, Coussens LM, Balkwill FR (2011). B regulatory cells and the tumor-promoting actions of TNF-alpha during squamous carcinogenesis. Proceedings of the National Academy of Sciences of the United States of America.

[75] Tretter T, Venigalla RK, Eckstein V, Saffrich R, Sertel S, Ho AD, Lorenz HM (2008). Induction of CD4+ T-cell anergy and apoptosis by activated human B cells. Blood.

[76] Rosser EC, Mauri C (2015). Regulatory B cells: origin phenotype and function. Immunity.

[77] Biswas SK, Mantovani A (2010). Macrophage plasticity and interaction with lymphocyte subsets: cancer as a paradigm. Nature immunology.

[78] Bodogai M, Moritoh K, Lee-Chang C, Hollander CM, Sherman-Baust CA, Wersto RP, Araki Y, Miyoshi I, Yang L, Trinchieri G, Biragyn A (2015). Immunosuppressive and prometastatic functions of myeloid-derived suppressive cells rely upon education from tumor-associated B cells. Cancer research.

[79] Xing C, Ma N, Xiao H, Wang X, Zheng M, Han G, Chen G, Hou C, Shen B, Li Y, Wang R (2015). Critical role for thymic CD19+CD5+CD1dhiIL-10+ regulatory B cells in immune homeostasis. Journal of leukocyte biology.

[80] Wang Z, Cheng Q, Tang K, Sun Y, Zhang K, Zhang Y, Luo S, Zhang H, Ye D, Huang B (2015). Lipid mediator lipoxin A4 inhibits tumor growth by targeting IL-10-producing regulatory B (Breg) cells. Cancer letters.

[81] Zheng M, Xing C, Xiao H, Ma N, Wang X, Han G, Chen G, Hou C, Shen B, Li Y, Wang R (2014). Interaction of CD5 and CD72 is involved in regulatory T and B cell homeostasis. Immunological investigations.

[82] Biragyn A, Lee-Chang C, Bodogai M (2014). Generation and identification of tumor-evoked regulatory B cells. Methods in molecular biology.

[83] Zhang Y, Morgan R, Podack ER, Rosenblatt J (2013). B cell regulation of anti-tumor immune response. Immunologic research.

[84] Zhang Y, Morgan R, Chen C, Cai Y, Clark E, Khan WN, Shin SU, Cho HM, Al Bayati A, Pimentel A, Rosenblatt JD (2016). Mammary-tumor-educated B cells acquire LAP/TGF-beta and PD-L1 expression and suppress anti-tumor immune responses. International immunology.

[85] Shalapour S, Font-Burgada J, Di Caro G, Zhong Z, Sanchez-Lopez E, Dhar D, Willimsky G, Ammirante M, Strasner A, Hansel DE, Jamieson C, Kane CJ, Klatte T (2015). Immunosuppressive plasma cells impede T-cell-dependent immunogenic chemotherapy. Nature.

[86] Schietinger A, Greenberg PD (2014). Tolerance and exhaustion: defining mechanisms of T cell dysfunction. Trends in immunology.

[87] Tang A, Dadaglio G, Oberkampf M, Di Carlo S, Peduto L, Laubreton D, Desrues B, Sun CM, Montagutelli X, Leclerc C (2016). B cells promote tumor progression in a mouse model of HPV-mediated cervical cancer. International journal of cancer Journal international du cancer.

[88] Affara NI, Ruffell B, Medler TR, Gunderson AJ, Johansson M, Bornstein S, Bergsland E, Steinhoff M, Li Y, Gong Q, Ma Y, Wiesen JF, Wong MH (2014). B cells regulate macrophage phenotype and response to chemotherapy in squamous carcinomas. Cancer cell.

[89] Hojyo S, Sarkander J, Manne C, Mursell M, Hanazawa A, Zimmel D, Zhu J, Paul WE, Fillatreau S, Lohning M, Radbruch A, Tokoyoda K (2016). B cells negatively regulate the establishment of CD49b(+)T-bet(+) resting memory T helper cells in the bone marrow. Frontiers in immunology.

[90] Novinger LJ, Ashikaga T, Krag DN (2015). Identification of tumor-binding scFv derived from clonally related B cells in tumor and lymph node of a patient with breast cancer. Cancer immunology immunotherapy.

[91] Woo JR, Liss MA, Muldong MT, Palazzi K, Strasner A, Ammirante M, Varki N, Shabaik A, Howell S, Kane CJ, Karin M, Jamieson CA (2014). Tumor infiltrating B-cells are increased in prostate cancer tissue. Journal of translational medicine.

[92] Vivanco BC, Viana JD, Perez EC, Konno FT, Guereschi MG, Xander P, Keller AC, Lopes JD (2014). B-1 cells promote immunosurveillance against murine melanoma in host absence of CCR5: new perspective in autologous vaccination therapy. Immunobiology.

[93] Ammirante M, Luo JL, Grivennikov S, Nedospasov S, Karin M (2010). B-cell-derived lymphotoxin promotes castration-resistant prostate cancer. Nature.

[94] Ammirante M, Kuraishy AI, Shalapour S, Strasner A, Ramirez-Sanchez C, Zhang W, Shabaik A, Karin M (2013). An IKKalpha-E2F1-BMI1 cascade activated by infiltrating B cells controls prostate regeneration and tumor recurrence. Genes & development.

[95] Andreu P, Johansson M, Affara NI, Pucci F, Tan T, Junankar S, Korets L, Lam J, Tawfik D, DeNardo DG, Naldini L, de Visser KE, De Palma M (2010). FcRgamma activation regulates inflammation-associated squamous carcinogenesis. Cancer cell.

[96] de Visser KE, Korets LV, Coussens LM (2005). De novo carcinogenesis promoted by chronic inflammation is B lymphocyte dependent. Cancer cell.

[97] Liu Y, Liu D, Wang C, Liao Q, Huang J, Jiang D, Shao W, Yin CC, Zhang Y, Lee G, Qiu X (2015). Binding of the monoclonal antibody RP215 to immunoglobulin G in metastatic lung adenocarcinomas is correlated with poor prognosis. Histopathology.

